# Supplementary material for: The impact of paediatric epilepsy and co-occurring neurodevelopmental disorders on functional brain networks in wake and sleep
Source: PLoS One. 2024 Aug 26;19(8):e0309243. doi: 10.1371/journal.pone.0309243 (PMC11346934; doi:10.1371/journal.pone.0309243)
Supplement: S1 File — (PDF) [file pone.0309243.s001.pdf]

Supporting Information

Network markers

Mean Degree (MD): The weighted degree for a node i is given by $\sum_{j=1}^{N} a_{ij}$, and the weighted mean degree is given by $\frac{1}{N}\sum_{i=1}^{N}\sum_{j=1}^{N} a_{ij}$.

Degree standard deviation (DStd): The weighted-degree standard deviation is given by $\sqrt{\frac{1}{N-1}\sum_{i=1}^{N}|D_i - MD|^2}$, where $D_i = \sum_{j=1}^{N} a_{ij}$ is the weighted degree of node i.

Average local clustering coefficient (ALCC): The weighted local clustering coefficient for a node i is given by:

$$C_i = \frac{1}{k_i(k_i-1)}\sum_{j=1}^{N}\sum_{k=1}^{N}\left(a_{ij}a_{jk}a_{ki}\right)^{\frac{1}{3}},$$

where C_i is defined to be 0 if $k_i \leq 1$. k_i is the unweighted degree of node i, given by $k_i = |\{a_{ij}|\ a_{ij} > 0\}|$ (the size of the set containing all nonzero edges between node i and the other nodes). The average local clustering coefficient is given by:

$$ALCC = \frac{1}{N}\sum_{i=1}^{N} C_i.$$

Global Efficiency (GE): Let $L_{ij} = \frac{1}{a_{ij}}$ denote the pairwise length between any two nodes i and j (smaller weights result in larger lengths, and if $a_{ij} = 0$ then the pairwise length is infinite). Let W_{ij} denote the set of finite sequences, or walks, $w = (L_{ik_1}, \ldots L_{k_m k_{m+1}}, \ldots, L_{k_M j})$ from node i to j, where if $M = 0$, then the walk is given by $\left(L_{ij}\right)$, if $M = 1$, then the walks are given by $(L_{ik_1}, L_{k_1 j})$, for $k_1 \in \{1,..,N\}\backslash\{i,j\}$, and so on. Define the function: $L(w) = \sum w$ for all $w \in W_{ij}$, which gives the length of each walk. Then the distance between i and j is given by $d_{ij} = L(\overline{w})$, for any $\overline{w} \in \{w \in W_{ij}|L(w^*) \geq L(w)\ \forall\ w^* \in W_{ij}\}$. In other words, d_{ij} is given by the shortest path length between the two nodes. If $L(w) = \infty\ \forall\ w \in W_{ij}$, then $d_{ij} = \infty$ (node i and j are not in the same connected component). The global efficiency is given by:

$$GE = \frac{1}{N(N-1)}\sum_{i=1}^{N}\sum_{j\neq i}^{N}\frac{1}{d_{ij}},$$

where if $d_{ij} = \infty$ then $\frac{1}{d_{ij}} = 0$.

Generally, the network markers DStd, GE and AWCC are correlated with MD. In order to correct for this effect, these markers were normalized by their average value calculated over 500 randomized versions of the original network.

Stage (# of epochs)	Diagnosis	Subjects (Cont/Epi)	Sex ratio (F/M)	Median age (Cont/Epi)	Cont-Epi age diff. (p-value)
Wake (31)	Cont	9	2 (6/3)	9	0.84
	Epi	22	0.47 (7/15)	9.5	
N1 (27)	Cont	7	2.5 (5/2)	8	0.18
	Epi	20	0.43 (6/14)	10	
N2 (21)	Cont	6	2 (4/2)	7.5	0.46
	Epi	15	0.25 (3/12)	9	

Table S1: Number of epochs in each stage for epilepsy diagnosis, sex ratio (female/male), and age distribution.

Subject	Sex	Age	Epilepsy	SCQ	Conners	Wake	Sleep N1	Sleep N2
PCB11	Male	13	Focal	9	4	Yes	Yes	Yes
PCB12	Male	9	Rolandic	6	12	No	Yes	Yes
PCB14	Male	8	Encephalopathy	27	8	Yes	Yes	Yes
PCB15	Male	7	No Epilepsy	2	3	Yes	Yes	Yes
PCB16	Male	9	Generalized	25	17	Yes	No	No
PCB17	Female	8	No Epilepsy	8	8	Yes	Yes	Yes
PCB19	Female	12	No Epilepsy	24	19	Yes	Yes	Yes
PCB20	Female	9	No Epilepsy	11	4	Yes	No	No
PCB25	Male	14	Focal	21	18	Yes	Yes	Yes
PCB27	Female	9	No Epilepsy	27	17	Yes	Yes	Yes
PCB28	Female	7	No Epilepsy	4	9	Yes	Yes	Yes
PCB29	Male	15	Rolandic	10	0	Yes	Yes	Yes
PCB3	Male	4	No Epilepsy	9	15	No	Yes	Yes
PCB30	Male	14	Generalized	9	10	Yes	Yes	Yes
PCB31	Male	12	Focal	16	4	Yes	Yes	No
PCB32	Male	9	Focal	8	11	Yes	Yes	Yes
PCB34	Male	6	Encephalopathy	12	13	Yes	Yes	Yes
PCB36	Female	10	Focal	3	0	Yes	Yes	No
PCB38	Female	12	Generalized	10	12	Yes	Yes	No
PCB40	Male	13	No Epilepsy	21	17	Yes	No	No
PCB44	Female	11	Generalized	6	1	Yes	Yes	No
PCB45	Male	15	No Epilepsy	0	0	Yes	No	No
PCB46	Male	10	Focal	18	20	No	Yes	Yes
PCB47	Male	11	Generalized	14	11	Yes	Yes	No
PCB5	Male	12	Focal	11	7	Yes	Yes	Yes
PCB50	Female	10	No Epilepsy	5	3	Yes	Yes	No
PCB51	Male	11	Focal	9	2	Yes	No	No
PCB52	Male	6	Focal	5	14	Yes	Yes	Yes
PCB58	Female	7	Focal	3	11	Yes	Yes	Yes
PCB61	Male	6	Focal	4	20	Yes	Yes	Yes
PCW3	Female	7	Generalized	2	18	Yes	Yes	Yes
PCW4	Female	5	Generalized	7	4	Yes	No	Yes
RPB1	Male	8	Rolandic	10	0	Yes	No	No
RPB6	Female	9	Rolandic	17	1	Yes	Yes	No

Table S2: Subjects metadata, indicating sex, age, epilepsy diagnosis, SCQ and Conners raw score, and for which stages a suitable clean EEG epoch has been identified (see Methods for details).

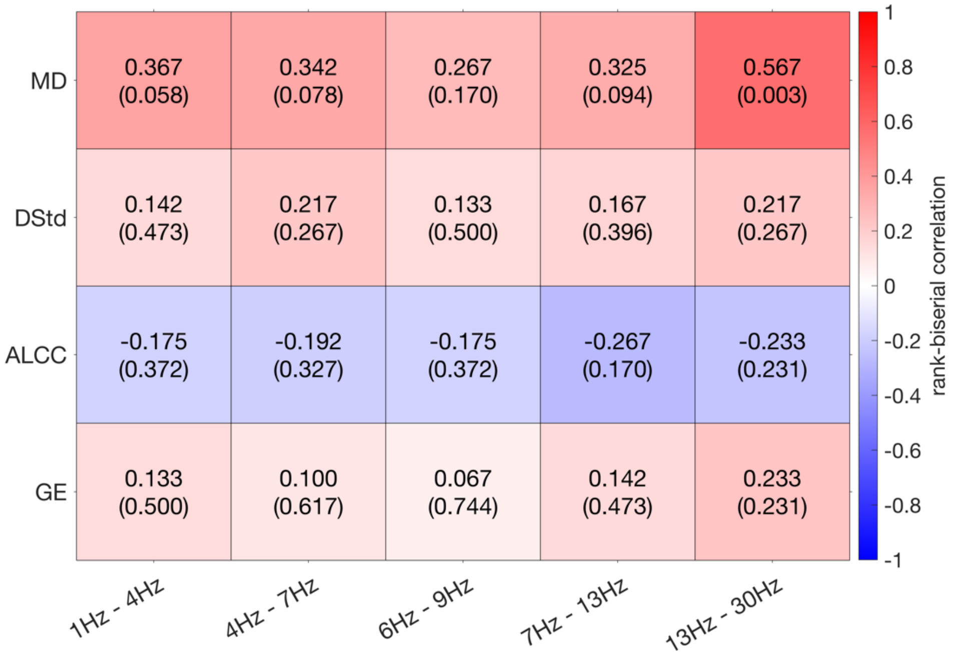

Figure S1: Rank-biserial correlation (and p-value) calculated for the comparison between Cont and Epi (a positive correlation means Cont > Epi) using the mean degree (MD), degree standard deviation (DStd), average weighted clustering coefficient (AWCC), and global efficiency (GE). These results were calculated using wake epochs.

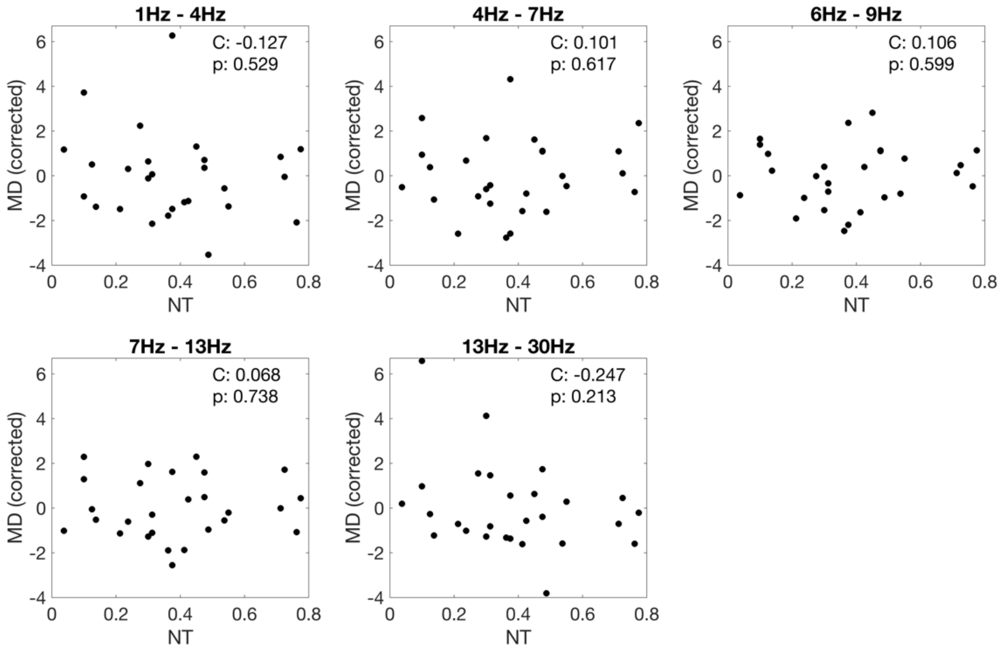

Figure S2: Spearman correlation and p-value (C and p) between neurodevelopmental trait index and mean degree (MD) corrected for age, sex, and epilepsy diagnosis. MD calculated using N1 epochs.

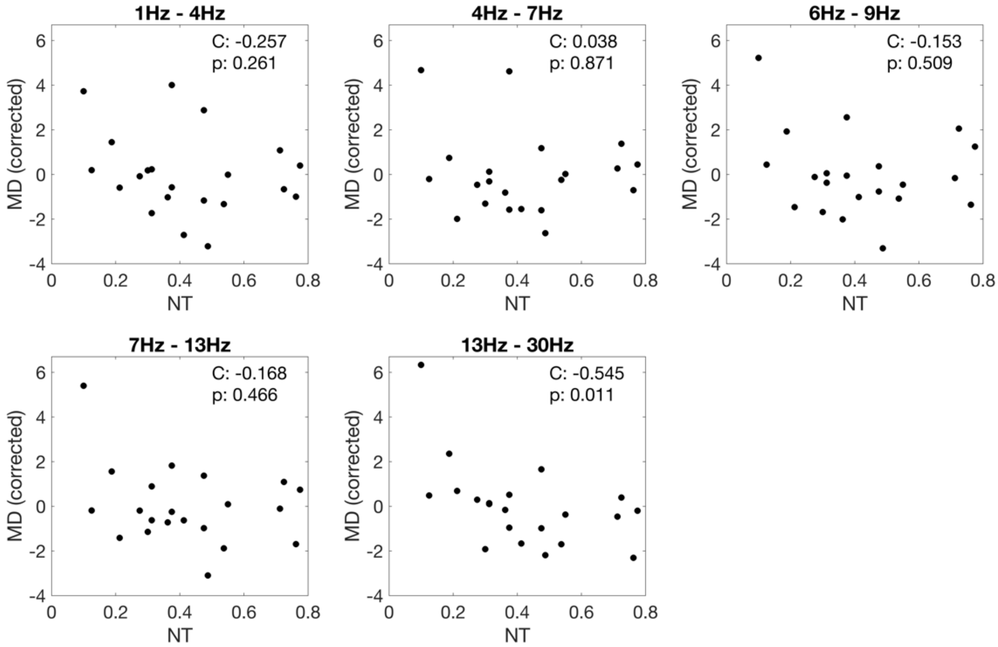

Figure S3: Spearman correlation and p-value (C and p) between neurodevelopmental trait index and mean degree (MD) corrected for age, sex, and epilepsy diagnosis. MD calculated using N2 epochs.

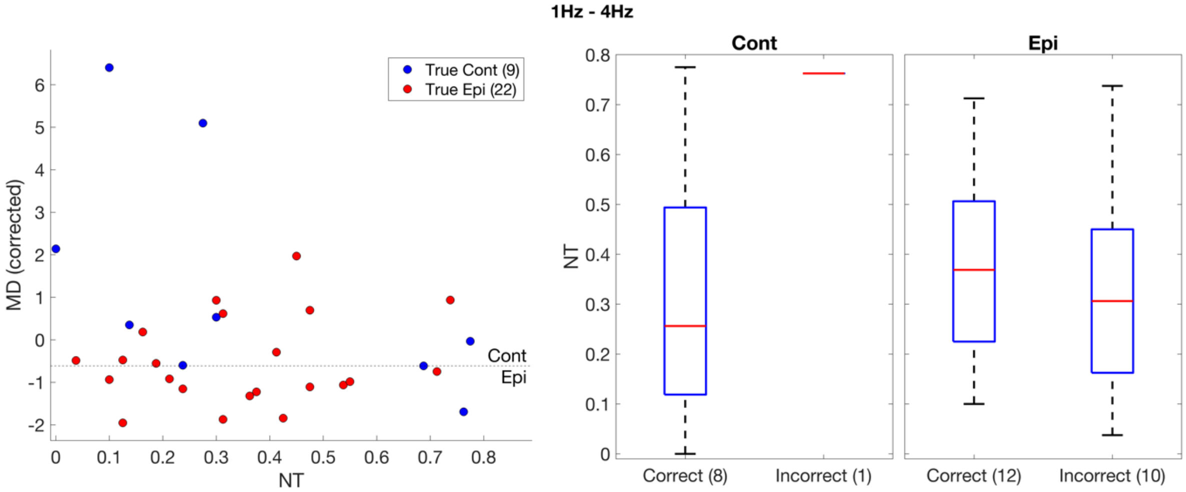

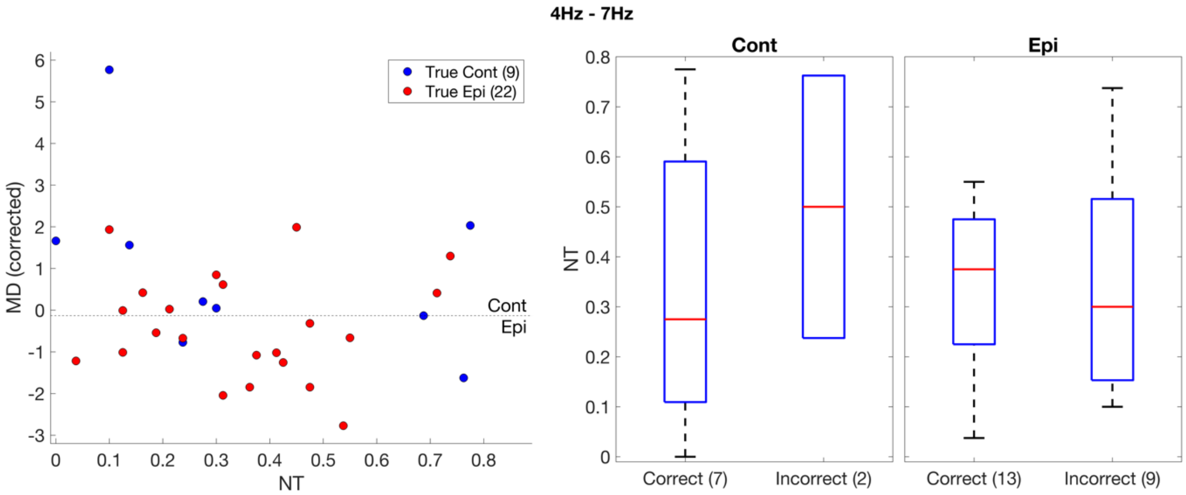

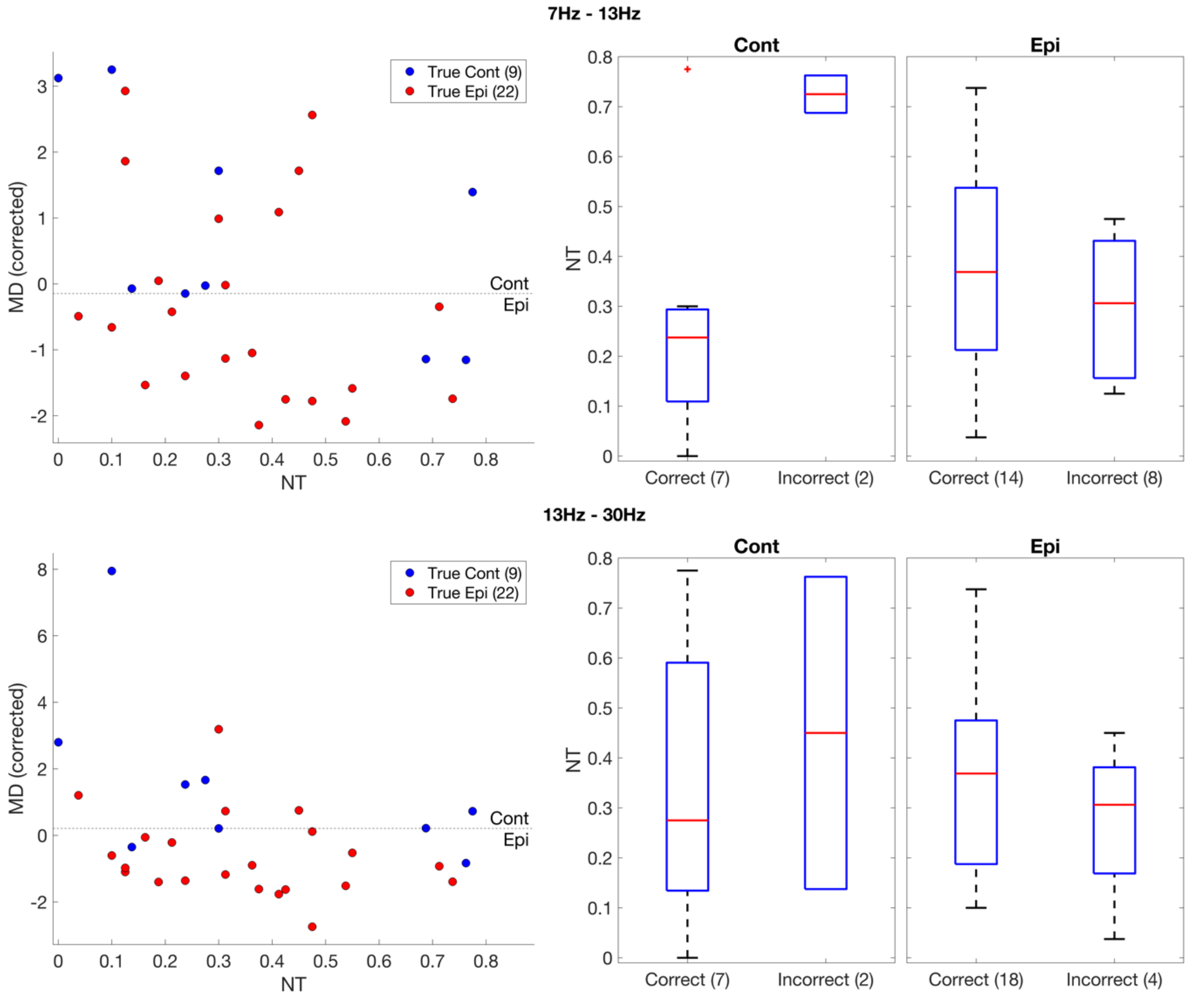

Figure S4: (Left) mean degree corrected for age, calculated using wake epochs, as a function of neurodevelopmental traits. Cont (Epi) are shown in blue (red). The dashed line represents the threshold of optimal balanced accuracy, for the separation between Cont and Epi. (Right) Comparison between neurodevelopmental traits of subjects classified correctly (Cont > threshold / Epi < threshold) and incorrectly (Cont < threshold / Epi > threshold). Each row of plots represents a different frequency band, indicated in the title.
